# Transmitted drug resistance mutations and subtype diversity amongst HIV-1 sero-positive voluntary blood donors in Accra, Ghana

**DOI:** 10.1186/s12985-020-01386-y

**Published:** 2020-07-24

**Authors:** Billal Musah Obeng, Evelyn Yayra Bonney, Lucy Asamoah-Akuoko, Nicholas Israel Nii-Trebi, Gifty Mawuli, Christopher Zaab-Yen Abana, Kwamena William Coleman Sagoe

**Affiliations:** 1 Department of Virology, Noguchi Memorial Institute for Medical Research (NMIMR), University of Ghana, Accra, Ghana; 2 Department of Medical Microbiology, School of Medicine and Dentistry, University of Ghana, Accra, Ghana; 3 Department of Research, National Blood Service, Accra, Ghana; 4 Department of Medical Laboratory Sciences, School of Biomedical and Allied Health Sciences, University of Ghana, Accra, Ghana

**Keywords:** HIV-1, Subtype diversity, Transmitted drug resistance, Blood donors

## Abstract

**Background:**

Detection of HIV-1 transmitted drug resistance (TDR) and subtype diversity (SD) are public health strategies to assess current HIV-1 regimen and ensure effective therapeutic outcomes of antiretroviral therapy (ART) among HIV-1 patients. Globally, limited data exist on TDR and SD among blood donors. In this study, drug resistance mutations (DRMs) and SD amongst HIV-1 sero-positive blood donors in Accra, Ghana were characterized.

**Methods:**

Purposive sampling method was used to collect 81 HIV sero-positive blood samples from the Southern Area Blood Center and confirmed by INNO-LIA as HIV-1 and/or HIV-2. Viral RNA was only extracted from plasma samples confirmed as HIV-1 positive. Complementary DNA (cDNA) was synthesized using the RNA as a template and subsequently amplified by nested PCR with specific primers. The expected products were verified, purified and sequenced. Neighbour-joining tree with the Kimura’s 2-parameter distances was generated with the RT sequences using Molecular Evolutionary Genetic Analysis version 6.0 (MEGA 6.0).

**Results:**

Out of the 81 plasma samples, 60 (74%) were confirmed as HIV-1 sero-positive by INNO-LIA HIVI/II Score kit with no HIV-2 and dual HIV-1/2 infections. The remaining samples, 21 (26%) were confirmed as HIV sero-negative. Of the 60 confirmed positive samples, (32) 53% and (28) 47% were successfully amplified in the RT and PR genes respectively. Nucleotide sequencing of amplified samples revealed the presence of major drug resistance mutations in two (2) samples; E138A in one sample and another with K65R. HIV-1 Subtypes including subtypes A, B, CRF02_AG and CRF09_cpx were found.

**Conclusion:**

This study found major drug resistance mutations, E138A and K65R in the RT gene that confer high level resistance to most NNRTIs and NRTI respectively. CRF02_AG was most predominant, the recorded percentage of subtype B and the evolutionary relationship inferred by phylogenetic analysis may suggest possible subtype importation. However, a more prospective and detailed analysis is needed to establish this phenomenon. The data obtained would inform the selection of drugs for ART initiation to maximize therapeutic options in drug-naïve HIV-1 patients in Ghana.

## Background

The HIV global burden is estimated at 36.9 million infected persons with approximately 25 million living in Sub-Saharan Africa [1]. HIV-1 infection was first detected in Ghana in 1986 and has since been responsible for numerous deaths of both children and adults [2]. Currently, the prevalence of HIV-1 in Ghana is estimated to be 1.7% in 2017 [3].

A major therapeutic intervention to the HIV pandemic has been the introduction and access to antiretroviral therapy (ART) [4]. This has reduced HIV-related morbidity and mortality rates and increased life expectancy of infected individuals [5]. However, therapeutic success of these ARTs is reduced as a result of emergence of HIV-1 DRMs, viral SD and spontaneously generated polymorphisms due to immune pressure in patients receiving ARTs [6]. The absence of genotypic drug resistance monitoring may lead to patients harboring viruses with DRM which could be transmitted to new hosts, a phenomenon described as transmitted drug resistance (TDR) [7].

TDR is an important public health concern due to increased risk of virologic failure when ART is initiated [8]. Systematic studies have suggested higher rates of TDR (11–23%) in Europe, America, and Australia, as compared to countries where scale-up of ART is ongoing [9–11]. In these studies, the prevalence of TDR in nucleoside reverse transcriptase inhibitors (NRTI), non-nucleotide reverse transcriptase inhibitors (NNRTI) and protease inhibitors (P1), were 7.4, 3.4 and 3% respectively.

Comprehensive care for people living with HIV/AIDS (PLHIV) in Ghana started in 2003 with first-line drugs including azidothymidine (AZT), stavudine (d4T), lamivudine (3TC), nevirapine (NVP) and efavirenz (EFV). Over the past decade, these drugs have expanded to include emtricitabine (FTC), tenofovir (TDF), and abacavir (ABC). Since its inception, ART has been scaled up to about 40%of HIV patients [3]. Limited data has shown that DRM and TDR in naïve PLHIV in Ghana are low [12–15]. A threshold survey among pregnant women in the administrative region where ART was first introduced in Ghana observed a TDR rate of < 5% [14].

Voluntary blood donors (VBD) provide a population base which is young and are more likely to have been recently exposed to HIV [16]. In order to understand the transmission dynamics among newly exposed people living with HIV (PLHIV), TDR in a cross-section of VBD needs to be assessed.

## Materials and methods

### Study design and setting

A cross-sectional study using purposive sampling method was conducted at the Southern Area Blood Center (SABC), Ghana from August 2016 to February 2017. The center is a satellite facility of the National Blood Service, Ghana (NBS). It is mandated to collect, screen, and distribute safe blood to various hospitals and clinics in the southern part of Ghana. The NBS recruits VBD using a questionnaire to first assess their behavioral risk factors and health status to ascertain their suitability to donate blood. Upon successful recruitment and blood donation, the blood is screened for HBsAg, HCV antibodies, HIV and antibodies for *Treponema pallidum* as procedures for safe blood. The test algorithm for HIV-1 at the blood bank is by the detection of p24 antigen using the HIV (Ag/Ab) 4^thGen^ (Fortress Diagnostics Limited, Antrim, U.K). Currently, a more sensitive test tool such as the PCR and/or INNO-LIA is not employed.

### Ethics statement

Ethical approval was obtained from the Ethics and Protocol Review Committee of the College of Health Sciences, University of Ghana. Approval to select HIV positive samples was also obtained from the NBS.

### Study participants

A total of eighty-one (81) voluntarily donated blood samples that were rejected as been HIV sero-positive using the HIV (Ag/Ab) 4^thGen^ (Fortress Diagnostics Limited, Antrim, U.K) were used. A data extraction sheet was used in obtaining information on age and gender from the donors’ records upon approval from the SABC. Study numbers were assigned to anonymize the blood samples.

### Sample collection and confirmation

Plasma was obtained from the SABC and were transported in cold boxes with ice packs to the Virology Department of NMIMR and stored at − 30 °C until further processing. A confirmatory test (INNO-LIA™ HIV-I/II score, Fujirebio, Gent, Belgium) was done on all plasma samples following manufacturer’s protocol.

### RNA extraction and complementary DNA (cDNA) synthesis

Viral RNA was extracted using the QIAamp® viral RNA mini kit (QIAGEN, Hilden, Germany) following manufacturer’s protocol. A two-step reverse transcription method was used to generate complementary DNA (cDNA) of HIV-1 from extracted RNA using Transcriptor High Fidelity cDNA synthesis kit (Roche Diagnostics, Mannheim, Germany). An initial reaction mix of 2.0 μl random hexamer primer, 2.4 μl nuclease-free water and 7.0 μl of extracted RNA were incubated at 65 °C for 10 min and immediately placed on ice. A second reaction mix made up of 4.0 μl of 5X High fidelity reverse transcriptase (5X HFRT) buffer, 0.5 μl protector RNAase inhibitor, 2.0 μl deoxynucleotide triphosphates (dNTPs), 1.0 μl dithiothreitol (DTT) and 1.1 μl transcriptor HFRT enzyme was prepared. An aliquot of 8.6 μl of the second mix was added to the first reaction, thoroughly mixed and incubated at 45 °C for 30 min followed by 85 °C for 5 min.

### Polymerase Chain reaction (PCR) amplification

Nested PCR was done to separately amplify the protease (PR) and reverse transcriptase (RT) genes from the cDNA synthesized using the Expand High Fidelity^plus^ PCR kit (Roche Diagnostics, Mannheim, Germany) with specific primers and cycling conditions previously published [17]. In the first round, 5.0 μl of 5 × buffer with MgCl_2_, 0.5 μl of dNTPs, 1.0 μl each of forward and reverse primers, 0.25 μl of expand high fidelity polymerase and 12.25 μl of nuclease free water were added to 5.0 *μ* l of cDNA. In the second round of the PCR, 5.0 μl of 5X buffer with MgCl_2,_ 0.5 μl dNTPs, 0.5 μl of each of forward and reverse primers, 0.25 μl expand high fidelity polymerase and 15.25 μl nuclease-free water were added to 3.0 μl of round 1 product. A fragment of 463 base pairs (bp) and 887 bp for the PR and RT genes respectively, were generated and confirmed by agarose gel electrophoresis.

### Purification of PCR amplicons and cycle sequencing

Purification of nested PCR products was done using QIAquick PCR purification kit (QIAGEN, Hilden, Germany) following manufacturer’s protocol. Purified amplicons were eluted in 50 μl of elution buffer for cycle sequencing. The BigDye Terminator v3.1 Cycle Sequencing kit (Applied Biosystems, MA, U.S.A) was used to separately sequence the PR and RT genes of HIV-1 using primers and cycling conditions previously published [18]. A total reaction volume of 10 μl comprising of 2 μl each of a primer, BigDye terminator, BigDye terminator buffer, nuclease free water and purified PCR product was used. Sequenced products were purified using the Agencourt® CleanSEQ® Dye-Terminator Removal system (Agencourt Bioscience Corporation, U.S.A) following manufacturer’s protocol. The purified product was loaded onto the ABI 3130xl genetic analyzer (Applied Biosystems, MA, U.S.A) to generate sequence data for HIV-1 DRM and HIV subtype analyses.

### Sequence and phylogenetic analysis

Nucleotide sequences for each sample were assembled to form a contig using SeqManPro 13 (DNASTAR Incorporation, U.S.A). Consensus sequence obtained was aligned with an HIV reference sequence (B-HXB2-PRT_2253–3700) in BioEdit (http://www.mbio.ncsu.edu/Bioedit/bioedit.html). Sequences were submitted to the Stanford University HIV Drug Resistance Database (https://hivdb.stanford.edu/hivdb/by-sequences/) to assign subtypes detect HIV drug resistance mutations and. The subtypes were confirmed with the Los Alamos National Laboratory HIV Database (http://www.hiv.lanl.gov). Neighbor-joining tree with the Kimura’s 2-parameter distances was generated with the RT sequences using Molecular Evolutionary Genetic Analysis version 6.0 (MEGA 6).

### Data analysis

Statistical analysis was done using SPSS version 23 (Armonk, USA) to describe patients’ demographics using frequencies and percentages.

## Results

A total of eighty-one (81) HIV-1 sero-positive blood samples were selected from the blood bank for this study. The HIV status, age and gender characteristics of participants is summarized in Table 1. Majority (40%) were between the ages of 26 to 35 years with 46 to 55-year group being the least recorded (10%). Majority (74%) were males whilst 14% were females. Age and Gender information for 10 (12%) of the study samples were not available in the SABC donor records. Sixty (74%) of the samples were confirmed as HIV-1 positive, Twenty-one samples (26%) were found to be negative for HIV-1, HIV-2 or dual HIV-1/2 infection.

**Table 1:** Demographic characteristics and HIV status of study samples

Variable	Frequency (%)
**Age**	
≤ 25	19 (23)
26–35	32 (40)
36–45	12 (15)
46–55	8 (10)
N/A	10 (12)
**Gender**	
Male	60 (74)
Female	11 (14)
N/A	10 (12)
**HIV Status**	
HIV-1	60 (74)
HIV-2	0 (0)
HIV-1/2	0 (0)
HIV Negative	21 (26)
**Total**	81 (100)

Table 1 indicates the HIV status, age and gender characteristics of participants. Majority (40%) were between the ages of 26 to 35 years with 46 to 55-year group being the least recorded (10%). Majority (74%) were males whilst 14% were females. Demographics for 10 (12%) of the study samples were not available. Sixty (74%) of the samples were confirmed as HIV-1 positive, Twenty-one samples (26%) were found to be negative for HIV-1, HIV-2 or dual HIV-1/2 infection

Key: *N/A* Not Available, *HIV* Human Immunodeficiency Virus

Of the 60 samples confirmed reactive for HIV-1, 28 (47%) and 32 (53%) were successfully amplified for the PR and RT genes respectively. Of these, 12/28 (43%) and 20/32 (63%) were successfully sequenced for the PR and RT genes respectively. Eight samples had sequences for both the PR and RT genes. One (1) sample had minor DRM in the PR gene whilst 1 minor and 2 major DRMs were found in the RT gene of three (3) samples as shown in Table 2.

**Table 2:** Drug resistance mutations in samples successfully amplified and sequenced

Gene	Samples Amplified	Samples Sequenced	Samples with DRMs
**PR**	28	12	1 PI
**RT**	32	20	2 NRTIs 1 NNRTIs

Table 2shows the number of samples successfully amplified and sequenced in the PR and RT genes of HIV-1. Twenty-eight (50%) and 32 (53%) were successfully amplified for the PR and RT genes respectively. Of these, 12/28 (43%) and 20/32 (63%) were successfully sequenced for the PR and RT genes respectively. One (1) sample had minor DRM in the PR gene whilst 1 minor and 2 major DRMs were found in the RT gene of three (3) samples

Key: *PR* Protease, *RT* Reverse Transcriptase, *PI* Protease Inhibitor, *NRTIs* Nucleoside Reverse Transcriptase Inhibitors, *NNRTIs* Non-nucleoside Reverse Transcriptase Inhibitors

**Fig. 1:**
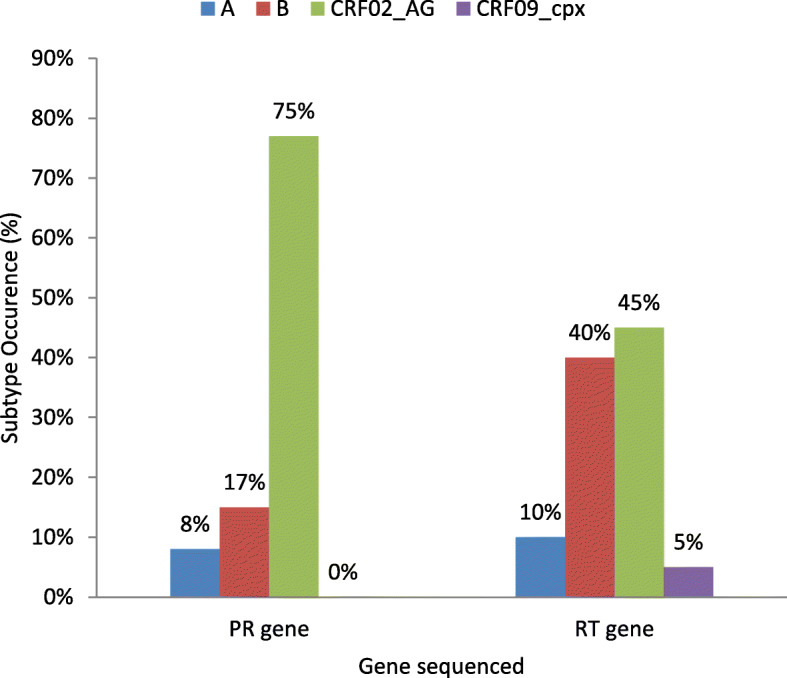
Percentage occurrence of HIV-1 subtypes in sequenced PR and RT genes. Figure 1 shows HIV-1 Subtype patterns in the PR and RT genes. PR and RT sequences were submitted online to the Stanford University HIV Drug Resistance Database (HIVdb). One (8%) was subtype A, 2 (17%) were subtype B and 9 (75%) were CRF02_AG. Of the 20 RT sequences, 2 (10%) were subtype A, 8 (40%) were subtype B, 9 (45%) were CRF02_AG and 1 (5%) was CRF09_cpx. Key: PR Protease, RT Reverse Transcriptase, CRF Circulating Recombinant Form

Out of the 12 PR sequences, 1 (8%) was subtype A, 2 (17%) were subtype B and 9 (75%) were CRF02_AG. Of the 20 RT sequences, 2 (10%) were subtype A, 8 (40%) were subtype B, 9 (45%) were CRF02_AG and 1 (5%) was CRF09_cpx as indicated in Fig. 1.

No major PR-related DRM was observed in the samples whilst major RT-related DRMs in two (2) samples were found; E138A in one sample and another with K65R. Drug resistance implications of the mutations found are shown in Table 3.

**Table 3:** Drug resistance mutations (DRMs) found and implications on available drug regimen

Study ID	Subtype	DRM		ARV
		PR	RT	
1	CRF02_AG	–	F77L	ABC, AZT, D4T, DDI, TDF, 3TC, FTC
2	CRF02_AG	L10F	–	FPV/r, IDV/r, NFV, LPV/r,
3	CRF02_AG	–	**E138A**	ETR, RPV
4	B		**K65R**	ABC, AZT, D4T, DDI, TDF

Table 3 Indicates the PR and RT related DRMs found and their drug resistance implications on available antiretroviral drug regimen. No major PR-related DRM was observed in the samples whilst major RT-related DRMs in two (2) samples were found; E138A in one sample and another with K65R

*NB:*[*A* Alanine, *D* Aspartate, *E* Glutamate, *F* Phenylalanine, *L* Leucine, *V* Valine, *K* Lysine, *R* Arginine, *FPV/r* Ritonavir-boosted fosamprenavir, *IDV/r* Ritonavir-boosted indinavir, *NFV* Nelfinavir, *LPV/r* Ritonavir-boosted lopinavir, *ABC* Abacavir, *AZT* Zidovudine, *D4T* Stavudine, *DDI* Didanosine, *TDF* Tenofovir, *3TC* Lamivudine, *FTC* Emtricitabine, *ETR* Etravirine, *RPV* Rilpivirine, *NVP* Nevirapine, *EFV* Efavirenz]

Key: *CRF* Circulating recombinant form

**Fig. 2:**
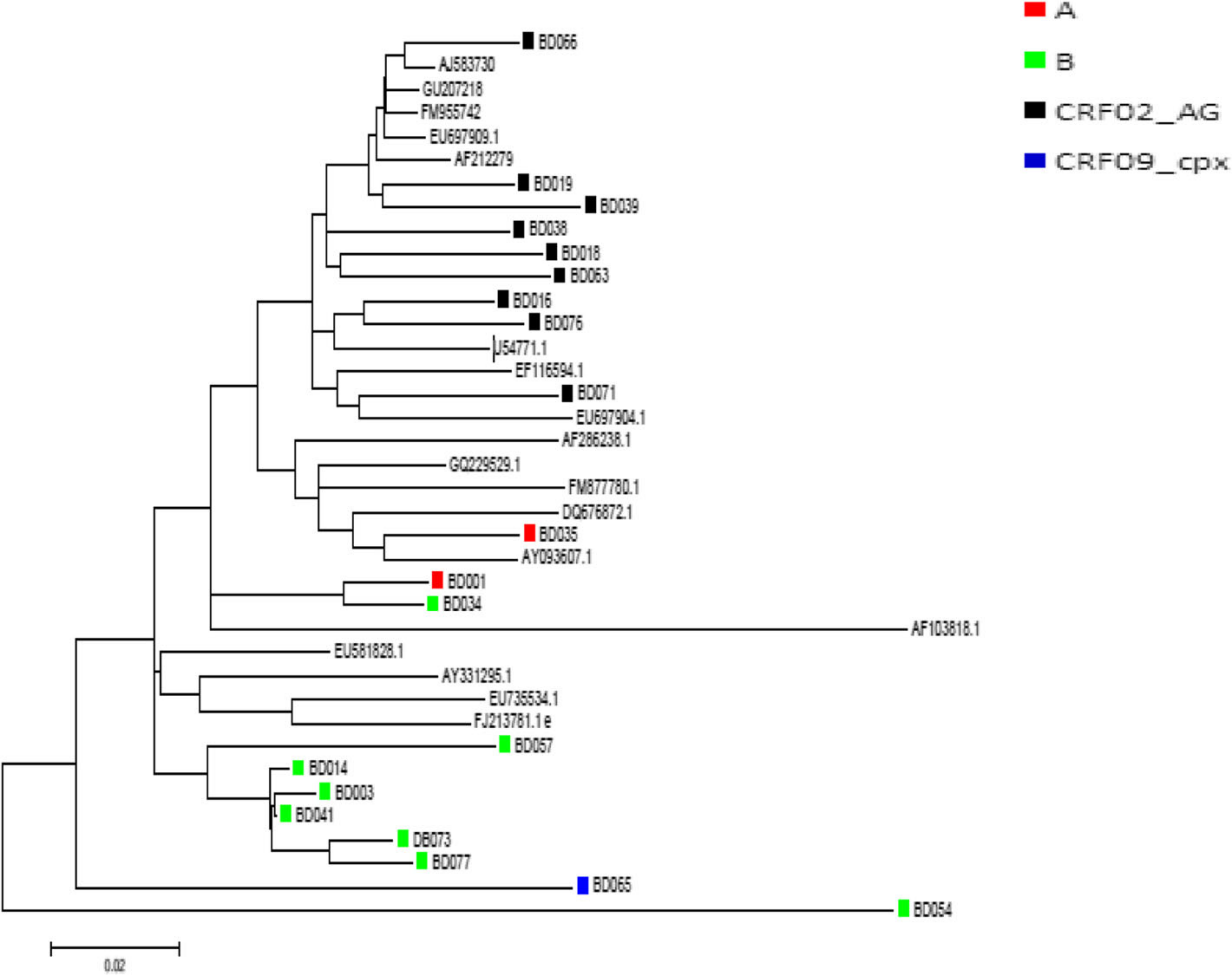
Phylogenetic analysis of RT sequences of HIV-1 in the study. Figure 2 shows the phylogenetic analysis of RT sequences using Neighbor-joining tree with the Kimura’s 2-parameter distances generated with Molecular Evolutionary Genetic Analysis tool version 6.0 (MEGA6). Study samples were labelled with coloured squares (red, green, blue and black squares indicating different subtypes) and references labelled with accession numbers. Study sequences clustered around reference sequences from Nigeria, Cameroon, Kingdom of Saudi Arabia, Democratic Republic of Congo, United States of America, Peru, Uruguay and Britain. Key: CRF Circulating Recombinant Form

Phylogenetic analysis using the Neighbour-joining tree with the Kimura’s 2-parameter distances was generated with the RT sequences using Molecular Evolutionary Genetic Analysis tool version 6.0 (MEGA6). Study samples were labelled with coloured squares (red, green, blue and black squares indicating different subtypes) and reference sequences labelled with accession numbers as shown in Fig. 2.

## Discussion

This study examined occurrence of drug-resistance mutations in apparently healthy blood donors who tested positive for HIV-1.The proportion of males to females in this study does not concord with HIV-1 trends. The same could be said for the distribution of HIV infection across the age groups. Although, the number of females infected with HIV nationwide is more than males [2], voluntary blood donors are usually males. Females are usually disqualified due to relatively lower hemoglobin (Hb) levels. This is partly because of monthly blood loss during menstruation and the toll of pregnancy. Moreover, it is physically active men usually between the ages of 25–50 years who volunteer to donate blood. Thus, these reasons could have accounted for the pattern seen in this study.

The predominance of CRF02_AG and absence of HIV-2 and dual HIV-1/2 infections agrees with studies reporting HIV-1 infection as the predominant HIV type in Ghana and CRF02_AG as the main subtype [19, 20]. The twenty (21) samples that were later confirmed to be HIV negative indicate the need for diagnostic methods with higher sensitivity at blood banks to minimize the probability of transfusing infected blood [10].

HIV-1 TDR occurs when recently infected individuals who are not exposed to antiretroviral drugs harbor drug resistant viruses. In this study, HIV-1 TDR is defined as the presence of at least one major HIV-1 DRM in a study participant. Two (2) major DRMs were found in two (2) samples sequenced in the RT gene (E138A and K65R) and none in the PR gene. Other mutations were also found, which do not confer drug resistance by themselves but only when they occur in combination with other mutations. The accessory and minor mutations found were F77L and L10F in RT gene and PR gene respectively.

The E138A mutation is a polymorphic mutation that occurs in an appreciable number of drug-naïve patients such as the population studied and confers resistance to etravirine (ETR) and rilpivirine (RPV), which are NNRTIs [21, 22]. In a similar study, E138A was found in ART-naïve pregnant women attending antenatal care in a teaching hospital in Accra, Ghana [23]. This mutation, has been shown to reduce susceptibility to ETR and RPV by 2-folds. The K65R mutation is found to reduce viral susceptibility to most NRTIs by approximately 2-fold and rather increase susceptibility to AZT and hence reduced viral replication with zidovudine-containing therapy [24].

The findings of this study are consistent with other studies previously conducted among drug naïve infected individuals, in which PI-associated DRM were rarely found [23, 25]. Generally, low level DRMs against PIs in the Ghanaian population could be attributed to the high genetic barrier of protease inhibitors and that the virus would have to mutate several times to develop resistance to such drugs. Additionally, sparing use of protease inhibitors reserved for use mostly in second-line regimen while majority of those on treatment are on reverse transcriptase inhibitors may have accounted for this phenomenon.

Viral sequence subtyping showed CRF02_AG as most predominant subtype in the study population. Other subtypes found were B, A and CRF09_cpx. This result is similar to some studies conducted in West Africa [26, 27] and in Ghana [19, 25]. The predominance of CRF02_AG has been associated with high viral infectivity and productivity, possible replicative fitness and high viral loads which favour viral transmission [19, 20]. Subtype B was the next predominant subtype and was relatively frequent compared to previous studies in Ghana [25]. Subtype B is most predominant in the Americas [28, 29] and Western Europe [30, 31].

The occurrence of HIV-1 subtypes in different geographical areas has been linked to socio-epidemiologic factors such as mobility and migration [32, 33]. These factors, however, cannot be confirmed for this population due to lack of data on residence and citizenship of the participants studied. However, phylogenetic analysis revealed evolutionary relationship of studied sequences with reference sequences from Nigeria, Cameroon, Kingdom of Saudi Arabia, Democratic Republic of Congo, United States of America, Peru, Uruguay and Britain.

The study observed low amplification rate of samples. This could be due to low viral loads in some samples. However, it is not entirely the reason for low amplification rate as samples with low viral loads were successfully amplified whilst others with higher loads were not amplified. Previous research shows that samples from patients with persistently low viral load could be genotyped by a nested PCR method [18, 24].

The inability to obtain peripheral blood mononuclear cells (PBMC) to amplify alongside the plasma could also account for a lower amplification rate. Amplification success with proviral DNA from PBMC was found to be relatively higher than viral RNA from plasma [22, 26]. Genotyping from plasma RNA and proviral DNA concurrently could have increased amplification success since some plasma samples may be amplified and not their PBMC samples and vice versa.

Despite these limitations, the study obtained data that is important for HIV management in Ghana.

## Conclusions

This study found major drug resistance mutations, E138A and K65R that respectively confer high level resistance to NNRTIs and NRTIs. Although, CRF02_AG was most predominant, the recorded percentage of subtype B and the evolutionary relationship inferred by phylogenetic analysis may suggest possible subtype importation. A more prospective and detailed analysis is needed to establish this phenomenon. The data obtained is useful for the selection of drugs for ART initiation to maximize therapeutic outcomes in drug-naïve HIV-1 patients in Ghana. Continuous surveillance for drug resistance mutations and subtype diversity in population groups is therefore imperative for effective ART outcomes.

## Data Availability

The data sets used and analysed during this study are available with the corresponding author on request.

## Abbreviations

μl: Microlitre
3TC: Lamivudine
ABC: Abacavir
ABI: Applied Biosystems Inc
ART: Antiretroviral Therapy
ARV: Antiretroviral
ATV: Atazanavir
AZT: Zidovudine
bp: Base pair
cDNA: Complementary DNA
CRF: Circulating Recombinant Form
D4T: Stavudine
DDI: Didanosine
DNA: Deoxyribonucleic Acid
DRM: Drug Resistance Mutation
DRV: Darunavir
ETR: Etravirine
FI: Fusion Inhibitors
FPV: Fosamprenavir
FTC: Emtricitabine
HBsAG: Hepatitis B Surface Antigen
HBV: Hepatitis B Virus
HCV: Hepatitis C Virus
HIV-1: Human Immunodeficiency Virus type 1
HIVdb: HIV Database
HIVDR: HIV Drug Resistance
IDV: Indinavir
II: Integrase Inhibitors
KBTH: Korle-Bu Teaching Hospital
LPV: Lopinavir
NBS: National Blood Service
NFV: Nelfinavir
NNRTI: Non-nucleoside Reverse Transcriptase Inhibitors
NRTI: Nucleoside Reverse Transcriptase Inhibitors
NVP: Nevirapine
PCR: Polymerase Chain Reaction
PI: Protease Inhibitors
PR: Protease
RNA: Ribonucleic Acid
RPV: Rilpivirine
RT: Reverse Transcriptase
SABC: Southern Area Blood Centre
SD: Subtype Diversity
TDF: Tenofovir
THFRT: Transcriptor High Fidelity Reverse Transcriptase
TDR: Transmitted Drug Resistance
UNAIDS: Joint United Nations Programme on HIV/AIDS
WHO: World Health Organization
